# Quiescence Entry, Maintenance, and Exit in Adult Stem Cells

**DOI:** 10.3390/ijms20092158

**Published:** 2019-05-01

**Authors:** Karamat Mohammad, Paméla Dakik, Younes Medkour, Darya Mitrofanova, Vladimir I. Titorenko

**Affiliations:** Department of Biology, Concordia University, 7141 Sherbrooke Street, West, SP Building, Room 501-13, Montreal, QC H4B 1R6, Canada; karamat.mohammad@concordia.ca (K.M.); pameladakik@gmail.com (P.D.); writetoyounes@gmail.com (Y.M.); mitrofanova_darya@hotmail.com (D.M.)

**Keywords:** cell cycle, cellular quiescence, mechanisms of quiescence maintenance, mechanisms of quiescence entry and exit, adult stem cells, metabolism, mitochondria, reactive oxygen species, cell signaling, proteostasis

## Abstract

Cells of unicellular and multicellular eukaryotes can respond to certain environmental cues by arresting the cell cycle and entering a reversible state of quiescence. Quiescent cells do not divide, but can re-enter the cell cycle and resume proliferation if exposed to some signals from the environment. Quiescent cells in mammals and humans include adult stem cells. These cells exhibit improved stress resistance and enhanced survival ability. In response to certain extrinsic signals, adult stem cells can self-renew by dividing asymmetrically. Such asymmetric divisions not only allow the maintenance of a population of quiescent cells, but also yield daughter progenitor cells. A multistep process of the controlled proliferation of these progenitor cells leads to the formation of one or more types of fully differentiated cells. An age-related decline in the ability of adult stem cells to balance quiescence maintenance and regulated proliferation has been implicated in many aging-associated diseases. In this review, we describe many traits shared by different types of quiescent adult stem cells. We discuss how these traits contribute to the quiescence, self-renewal, and proliferation of adult stem cells. We examine the cell-intrinsic mechanisms that allow establishing and sustaining the characteristic traits of adult stem cells, thereby regulating quiescence entry, maintenance, and exit.

## 1. Introduction

Cellular quiescence is a reversible state of a temporary cell cycle arrest that can be induced in both metazoans and unicellular eukaryotes as a response to some anti-mitogenic factors [1,2,3,4]. These factors include cell-nonautonomous, extrinsic environmental cues and cell-autonomous, intrinsic regulatory mechanisms [1,2,3,4]. In mammals, the temporary cell cycle arrest and quiescence entry occur before cells reach the growth factor-dependent “restriction” (R) point of the G_1_ phase [5,6]. In the budding yeast *Saccharomyces cerevisiae*, the nutrient-dependent “START A” point at the G_1_ phase of the cell cycle is believed to be evolutionarily related to the R point in mammals [6,7,8]. Notably, under certain conditions some unicellular and multicellular cells, eukaryotic organisms can undergo a temporary cell cycle arrest and enter the quiescent state not only from the G_1_ phase of the cell cycle, but also from the S, G_2_, or M phase [9,10,11,12,13,14,15,16,17,18,19]. Studies in budding yeast suggest that this is because the entry into quiescence is controlled not by (or not only by) the cell cycle regulation machinery, but by (or also by) the metabolic status of the cell at a certain cell cycle phase [17,18]. Once the cell cycle is arrested at the R or START A point, cells enter a reversible G_0_ phase of the cell cycle and become quiescent. In budding yeast and mammals, this reversible G_0_ state of quiescence is also called the “quiescence cycle” of cell oscillation between at least two functional states [1,2,3,4,20].

The entry of cells into the reversible G_0_ state of quiescence prevents their entry into the irreversible G_0_ state of senescence or the irreversible G_0_ state of terminal differentiation [2,3,4]. Of note, some “irreversibly arrested” senescent or terminally differentiated cells retain an intact (although silenced) mechanism for cell cycle re-entry, as they can resume proliferation in response to certain cell-extrinsic and cell-intrinsic factors [21,22,23,24,25]. Quiescent cells in the reversible G_0_ state do not divide, but rather retain the ability to re-enter the cell cycle and resume proliferation in response to certain pro-mitogenic factors, which include cell-extrinsic environmental signals and cell-intrinsic regulatory mechanisms [2,4]. Cellular quiescence is actively maintained by complex multiprotein networks and represents a collection of heterogeneous states in both multicellular and unicellular eukaryotes [2,3,4,26,27,28,29,30,31,32].

Populations of unicellular eukaryotic organisms (such as various yeast species) in the wild are always able to undergo a reversible switch between the states of cellular quiescence and proliferation; such a switch is controlled by nutrient availability and some other environmental factors [3,8,19,33,34,35,36]. Adult organisms in “lower” metazoan organisms (such as nematodes and fruit flies) and in “higher” metazoans (such as plants, mammals, and humans) contain several distinct types of quiescent cells; adult stem cells are among these quiescent cells in mammals and humans [2,37,38,39,40,41,42,43,44].

Quiescent adult stem cells in different mammalian tissues are long-lived [2,29,45,46]. This is because they can actively support their resistance to various stresses and toxicities [2,29,45,46]. This is also because, when stimulated, quiescent adult stem cells can often self-renew by dividing infrequently and asymmetrically to form a new quiescent stem cell and an actively dividing daughter progenitor cell; then, the daughter progenitor cell can advance through a hierarchically organized and tightly controlled series of events that yield one or more types of terminally differentiated cells [2,47,48,49,50,51]. Of note, quiescent adult stem cells can also sometime undergo two types of symmetric divisions, either a proliferation division (which yields two identical quiescent stem cells) or a differentiation division (which yields two differentiated cells) [49,52,53,54,55,56,57,58,59,60].

A body of evidence indicates that the abilities of quiescent adult stem cells to resist stresses, self-renew, and produce fully differentiated cells are crucial for tissue repair and regeneration, and are vital for the growth, development, and health of the adult body [2,19,48,61,62,63,64]. The number of quiescent adult stem cells and the efficiencies with which they resist stresses, self-renew, and produce fully differentiated cells declines with age [29,31,32,45,46,61,62,64,65,66,67,68,69,70,71,72,73,74,75,76,77,78,79,80,81]. Such an age-related numerical and functional decline of quiescent adult stem cells impairs their ability to balance quiescence with proliferation activity, and has been implicated in the pathophysiology of cancer and other aging-associated diseases in mammals and humans [19,29,31,32,45,46,48,61,62,64,66,67,69,70,72,73,74,75,76,77,79,80,81,82,83,84]. Some genetic, dietary, and pharmacological interventions can delay cellular and organismal aging and postpone the onset of aging-associated diseases by decelerating an age-related decline in the number and/or functionality of quiescent adult stem cells [2,19,29,31,32,45,46,48,61,62,64,65,66,67,68,69,70,71,72,73,74,75,76,77,78,79,80,81,82,83,84]. Aging-associated changes in the specialized cellular neighborhoods of adult stem cells, which are known as the stem cell niches, provide an essential contribution to an age-related decline in the abilities of adult stem cells to sustain quiescence, proliferation capacity, and differentiation potential [85,86,87,88,89,90,91]. This is because the stem cell niches in diverse tissues produce and release certain short-range molecular signals that are indispensable for maintaining the quiescence, self-renewal, proliferation capacity, differentiation potential, and functionality of neighboring adult stem cells [2,91,92,93]. Since the efficiencies with which the stem cell niches produce and release such transmissible molecular signals are either enhanced or weakened with age, the cell-nonautonomous mechanisms orchestrated by these niches critically contribute to the age-related weakening of various aspects of stem cell functionality [85,86,87,88,89,90,91].

Here, we examine the characteristic metabolic, signal transduction, gene expression, epigenetic, stress survival, and cell cycle regulation features of quiescent adult stem cells in mammals and humans. We explore cell-intrinsic mechanisms regulating quiescence entry, maintenance, and exit in these cells.

## 2. Common Traits of Quiescent Adult Stem Cells and Cell-Intrinsic Mechanisms That in These Cells Control Quiescence Entry, Maintenance, and Exit

**Figure 1:**
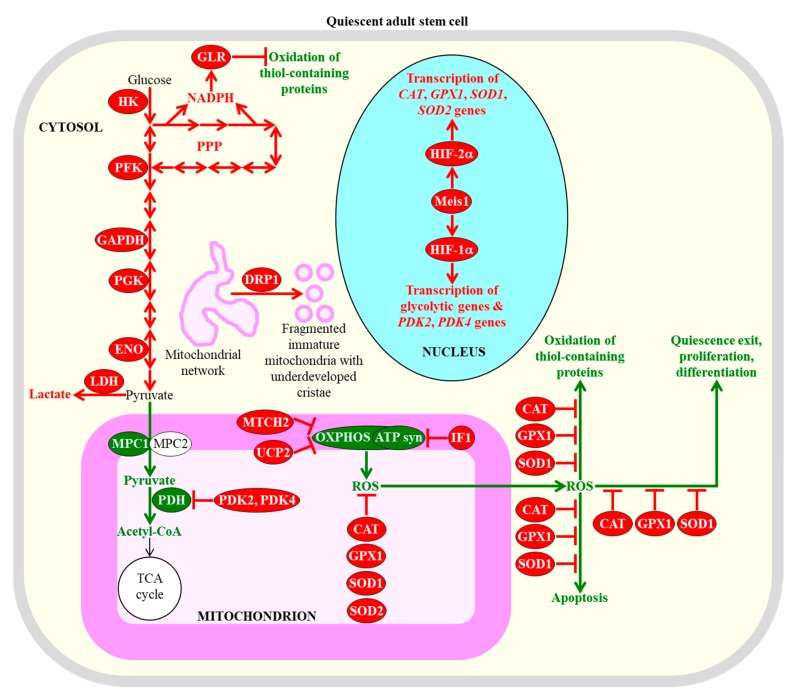
Some common metabolic features of adult stem cells are essential for the maintenance of their quiescence, number, proliferation potential, and differentiation ability. Among these metabolic features are carbohydrate metabolism mainly through aerobic glycolysis in the cytosol, suppressed carbohydrate oxidation in mitochondria, mitochondrial network fragmentation into globular and immature mitochondria with underdeveloped cristae, and stimulated ROS detoxification in several cellular locations. Enzymes, metabolites and processes whose activities, concentrations and rates are increased or decreased in quiescent adult stem cells (as compared to their fully differentiated progeny) are displayed in red or green color, respectively. The red one-way arrows and the red two-way arrows define irreversible and reversible (respectively) chemical reactions whose rates are increased in quiescent adult stem cells. The red inhibitory bars define inhibitory effects whose intensities are increased in quiescent adult stem cells. The green arrows define chemical reactions or processes whose rates or intensities are decreased in quiescent adult stem cells. The black arrow defines the irreversible chemical reaction whose rate is not changed in quiescent adult stem cells. See text for more details. Abbreviations: ATP syn, ATP synthase; CAT, catalase; DRP1, dynamin-related protein 1; ENO, enolase; GAPDH, glyceraldehyde 3-phosphate dehydrogenase; GLR, glutathione reductase; GPX1, glutathione peroxidase type 1; HIF-1α and HIF-2α, transcription factor hypoxia-inducible factors 1α and 2α, respectively; HK, hexokinase; IF1, inhibitory factor 1; LDH, lactate dehydrogenase; Meis1, myeloid ecotropic viral insertion site 1; MPC1 and MPC2, mitochondrial pyruvate carrier subunits 1 and 2; MTCH2, mitochondrial carrier homolog 2; OXPHOS, oxidative phosphorylation; PDH, pyruvate dehydrogenase; PDK2 and PDK4, pyruvate dehydrogenase kinases 2 and 4 (respectively); PFK, phosphofructokinase; PGK, phosphoglycerate kinase; PPP, pentose phosphate pathway; ROS, reactive oxygen species; SOD1 and SOD2, superoxide dismutases 1 and 2 (respectively); UCP2, uncoupling protein 2.

**Figure 2:**
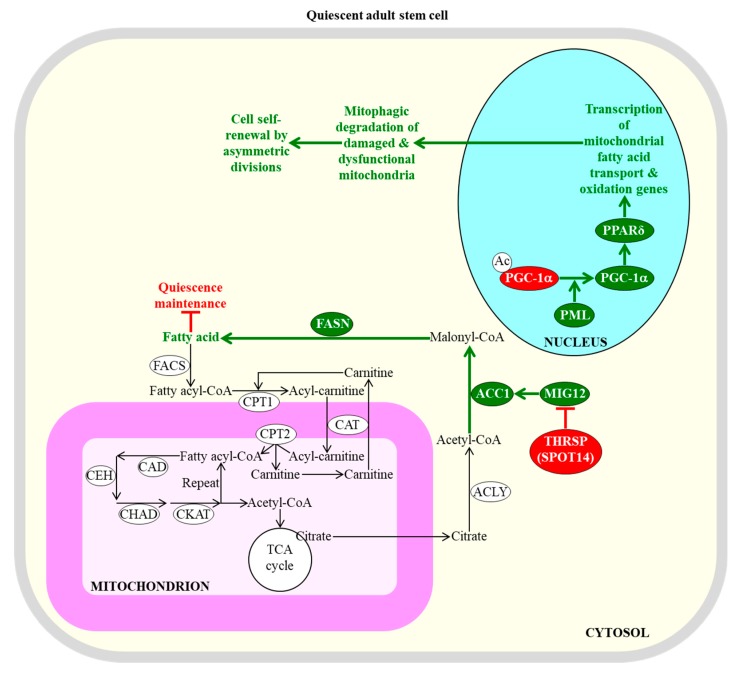
Mitochondrial β-oxidation of fatty acids and their synthesis in the cytosol of adult stem cells control the efficiency with which these cells can sustain quiescence or self-renew by asymmetric divisions. An inhibition of fatty acid synthesis in the cytosol is needed to sustain the quiescent state of adult neural stem cells. An activation of the transcription of nuclear genes that are involved in mitochondrial fatty acid transport and β-oxidation within adult stem cells is essential for the self-renewal of these cells by asymmetric divisions; such divisions lead to the formation of a new quiescent stem cell and an actively dividing daughter progenitor cell. Enzymes, metabolites, and processes whose activities, concentrations, and rates must be increased to maintain the quiescence of adult stem cells are displayed in red. Enzymes, metabolites, and processes whose activities, concentrations, and rates need be decreased to promote the self-renewal of adult stem cells by asymmetric divisions are displayed in green. The back arrows define chemical reactions whose rates are not essential for the maintenance of quiescence by adult stem cells. The green arrows define chemical reactions or processes whose rates or intensities must be decreased to maintain the quiescence of adult stem cells. The red inhibitory bars define inhibitory effects whose intensities must be increased to maintain the quiescence of adult stem cells. See text for more details. Abbreviations: Ac, acetyl group; ACC1, acetyl-CoA carboxylase 1; ACLY, ATP citrate lyase; CAD, acyl-CoA dehydrogenase; CAT, carnitine acylcarnitine translocase; CEH, enoyl-CoA hydratase; CHAD, hydroxyacyl-CoA dehydrogenase; CKAT, ketothiolase; CPT1 and CPT2, carnitine palmitoyltransferases 1 and 2 (respectively); FACS, fatty acyl-CoA synthase; FASN, fatty acid synthase; MIG12, midline-1-interacting G12-like protein; PGC-1α, peroxisome proliferator-activated receptor-gamma coactivator 1α; PML, promyelocytic leukaemia protein; PPARδ, peroxisome proliferator-activating receptor type δ; SPOT14, the 14th spot of proteins; THRSP, thyroid hormone-inducible hepatic protein.

**Figure 3:**
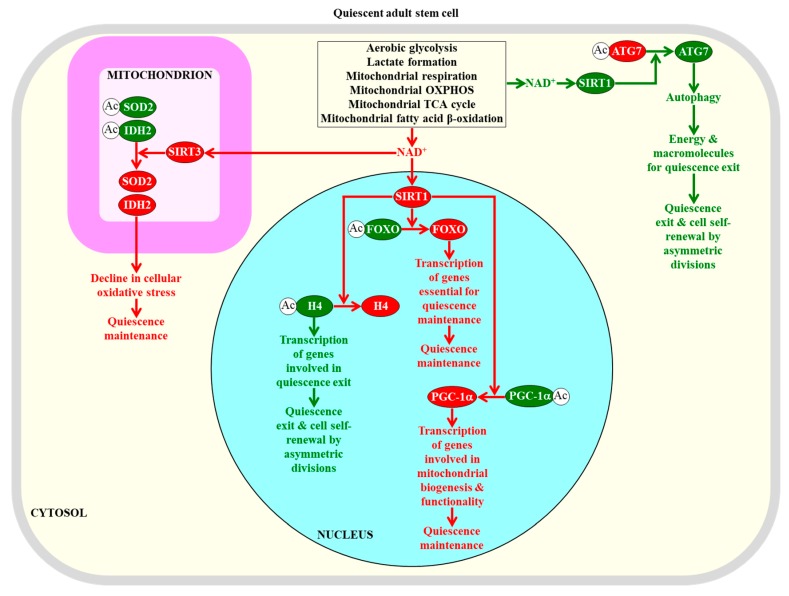
NAD^+^ concentration within adult stem cells defines how efficiently these cells can maintain quiescence or self-renew by undergoing asymmetric divisions. The NAD^+^-activated sirtuin SIRT1 in the nucleus is required for the maintenance of quiescence in adult stem cells, because this SIRT1 deacetylates histone H4 and the transcriptional factors FOXO and PGC-1α. The NAD^+^-activated sirtuin SIRT3 in mitochondria is essential for quiescence maintenance by adult stem cells, because this SIRT3 deacetylates and activates superoxide dismutase SOD2 and isocitrate dehydrogenase IDH2, both of which weaken cellular oxidative stress. The NAD^+^-activated sirtuin SIRT1 in the cytosol is required for the transition from quiescence to self-renewal by asymmetric divisions, because this SIRT1 deacetylates and activates the autophagy-related protein ATG7 to promote autophagy, which provides the energy and macromolecules required for such transitions. Proteins, metabolites, and processes whose activities, concentrations, and rates must be increased to maintain the quiescence of adult stem cells are displayed in red. Proteins, metabolites, and processes whose activities, concentrations, and rates must be increased to promote the self-renewal of adult stem cells by asymmetric divisions are displayed in green. The red arrows define processes whose intensities must be increased to maintain the quiescence of adult stem cells. The green arrows define processes whose intensities must be decreased to maintain the quiescence of adult stem cells. See text for more details. Abbreviations: Ac, acetyl group; ATG7, the autophagy-related protein 7; FOXO, transcriptional factors of the Forkhead family; H4, histone H4; IDH2, mitochondrial isocitrate dehydrogenase 2; OXPHOS, oxidative phosphorylation; PGC-1α, peroxisome proliferator-activated receptor-gamma coactivator 1α; SIRT1 and SIRT3, NAD^+^-dependent protein deacetylases sirtuin 1 and sirtuin 3 (respectively); SOD2, mitochondrial manganese superoxide dismutase.

**Figure 4:**
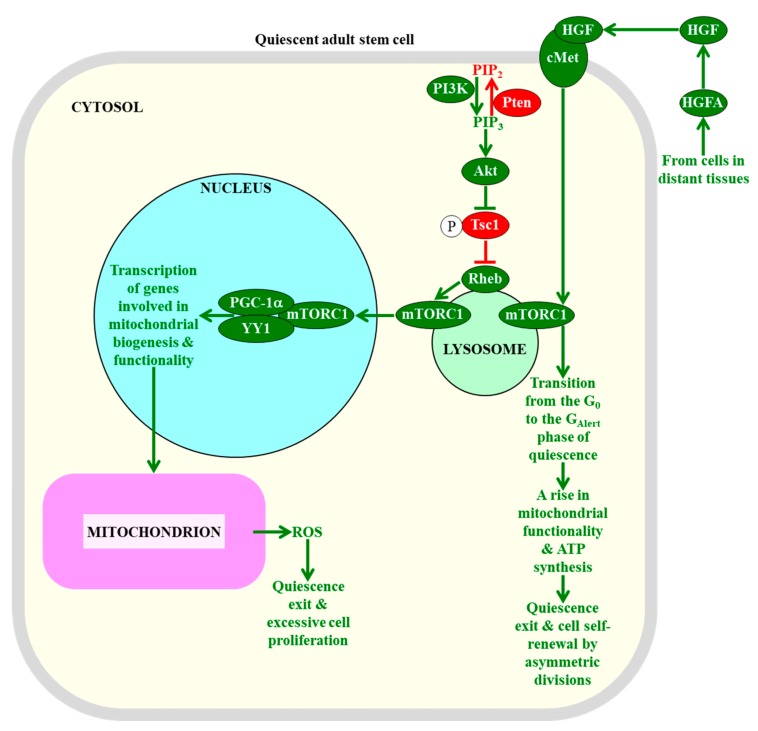
The intensity of information flow through the mTORC1-signaling pathway defines the fate of quiescent adult stem cells. A low intensity of mTORC1 signaling in adult stem cells decreases the extent of mitochondrial ROS production and release, thereby preventing the exit of these cells from the quiescent state and their excessive proliferation. An activation of mTORC1 signaling in response to hepatocyte growth factor, a cell-extrinsic pro-mitogenic signal, commits quiescent adult stem cells to cell cycle entry, proliferation, and differentiation. Proteins, metabolites, and processes whose activities, concentrations, and rates must be increased to maintain the quiescence of adult stem cells are displayed in red. Proteins, metabolites, and processes whose activities, concentrations, and rates need to be increased to promote the exit of adult stem cells from the state of quiescence are displayed in green. The red arrow defines the chemical reaction whose rate must be increased to maintain the quiescence of adult stem cells. The red inhibitory bar defines the inhibitory effect whose intensity must be increased to maintain the quiescence of adult stem cells. The green arrows define chemical reactions or processes whose rates or intensities must be increased to promote the exit of adult stem cells from the state of quiescence. See the text for more details. Abbreviations: Akt1, RAC-alpha serine/threonine protein kinase 1 or v-akt murine thymoma viral oncogene homolog 1; cMet, mesenchymal–epithelial transition factor; G_Alert_, the “alert” phase of quiescence; HGF, hepatocyte growth factor; HGFA, hepatocyte growth factor activator; mTORC1, the mammalian (or mechanistic) target of rapamycin complex 1; PGC-1α, peroxisome proliferator-activated receptor-gamma coactivator 1α; PIP_2_, phosphatidylinositol 4,5-bisphosphate; PIP_3_, phosphatidylinositol (3,4,5)-trisphosphate; PI3K, phosphoinositide 3-kinase; Pten, phosphatase and tensin homologue; Rheb, Ras homolog enriched in brain; ROS, reactive oxygen species; Tsc1, tuberous sclerosis 1; YY1, yin-yang 1 transcription factor.

**Figure 5:**
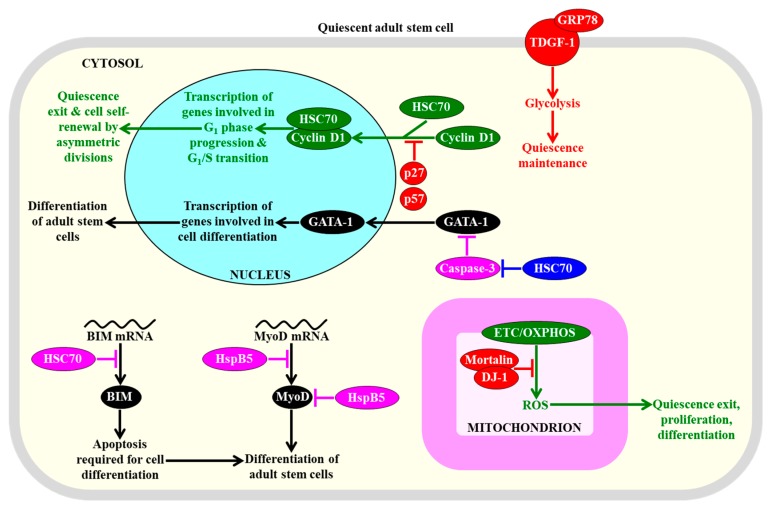
Several heat shock proteins (HSPs) play essential roles in preserving the quiescence, affecting the proliferation, and influencing the differentiation of adult stem cells. These HSPs control the nuclear import and degradation of some transcription factors, as well as the translation and stability of certain proteins that promote cell differentiation, glycolysis, and concentrations of mitochondrially-generated reactive oxygen species (ROS). Proteins, metabolites, and processes whose activities, concentrations, and rates must be increased to maintain the quiescence of adult stem cells are displayed in red. Proteins, metabolites, and processes whose activities, concentrations, and rates must be increased to promote the exit of adult stem cells from the state of quiescence are displayed in green. Proteins and processes whose activities and rates must be increased to promote the differentiation of adult stem cells are displayed in black. Proteins whose activities must be increased to suppress the differentiation of adult stem cells are displayed in pink. See text for more details. Abbreviations: BIM, Bcl-2-like protein 11; GATA-1, GATA-binding factor 1; GRP78, immunoglobulin heavy chain-binding protein homolog; HSC70, heat shock cognate 71 kDa protein; HspB5, alpha-crystallin B chain; MyoD, myoblast determination protein D; TDGF-1, teratocarcinoma-derived growth factor 1.

**Figure 6:**
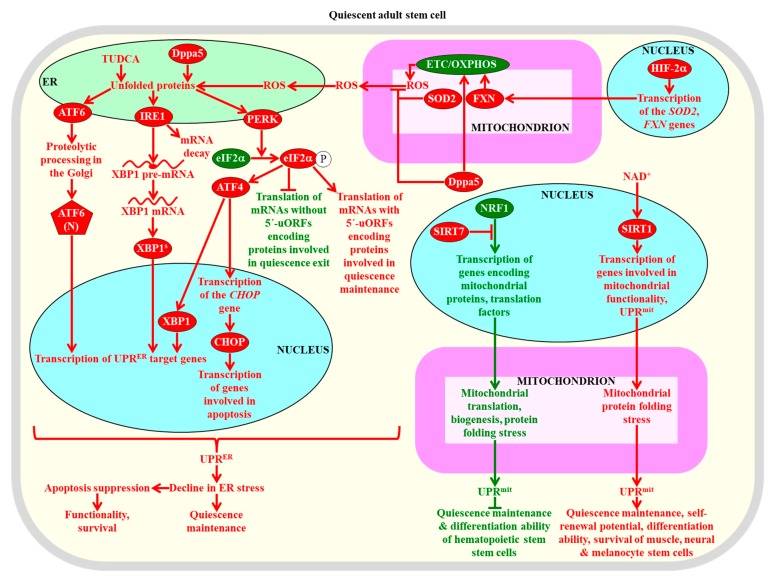
The unfolded protein response (UPR^ER^) and (UPR^mit^) systems of proteostasis restoration in adult stem cells are required for the maintenance of their quiescence, self-renewal proficiency, proliferation potential, differentiation competence, functionality, and viability. A stimulation of the ATF6 (activating transcription factor 6), IRE1 (inositol requiring enzyme 1), and PERK (PKR-like endoplasmic reticulum kinase) branches of the UPR^ER^ system is essential for the maintenance, self-renewal, functionality, and survival of adult stem cells. A SIRT7-dependent inhibition of the UPR^mit^ system is indispensable for sustaining the quiescence and differentiation capability of hematopoietic stem cells. A NAD^+^/SIRT1-dependent stimulation of the UPR^mit^ system is required for the maintenance of the quiescence, self-renewal potential, differentiation ability, and viability of muscle stem cells, neural stem cells, and melanocyte stem cells. Proteins, metabolites, and processes whose activities, concentrations, and rates must be increased to maintain the quiescence of adult stem cells are displayed in red. Proteins, metabolites, and processes whose activities, concentrations, and rates need to be increased to promote the exit of adult stem cells from the state of quiescence are displayed in green. See text for more details. Abbreviations: ATF4, activating transcription factor 4; ATF6(N), N-terminal cytosolic fragment; CHOP, C/EBP homologous protein; Dppa5, developmental pluripotency-associated 5 protein; eIF2α, eukaryotic translation initiation factor 2α; ER, the endoplasmic reticulum; ETC, electron transport chain; FXN, frataxin; HIF-2α, hypoxia-inducible factor 2α; NRF1, nuclear respiratory factor 1; OXPHOS, oxidative phosphorylation; ROS, reactive oxygen species; SIRT1 and SIRT7, sirtuin 1 and sirtuin 7 (respectively); SOD2, mitochondrial manganese superoxide dismutase; TUDCA, tauroursodeoxycholic acid; uORFs, upstream open reading frames; XBP1, X-box binding protein 1; XBP1^s^, X-box binding protein 1 translated as a protein product the spliced mRNA for XBP1.

Quiescent adult stem cells residing in different tissues share a discrete set of metabolic, signal transduction, gene expression, epigenetic, stress survival, and cell cycle regulation traits, all of which are distinct from those of the fully differentiated progeny of such cells [94,95,96,97]. Many of these common traits are actively maintained by quiescent adult stem cells and define their stress resistance, self-renewal potential, and regulated proliferation and differentiation routes; thus, adult stem cells have developed cell-autonomous, intrinsic regulatory mechanisms for quiescence entry, maintenance, and exit [28,94,95,96,97,98,99]. These traits and mechanisms are outlined below and schematically depicted in Figure 1, Figure 2, Figure 3, Figure 4, Figure 5 and Figure 6.

### 2.1. Common Metabolic Traits of Quiescent Adult Stem Cells Define Their Fate

Quiescent adult stem cells metabolize glucose and other carbohydrates mainly through aerobic glycolysis that yields pyruvate; instead of being transported to mitochondria, converted to acetyl-CoA, and incorporated into the mitochondrial tricarboxylic acid (TCA) cycle, this pyruvate is then transformed to lactate in the cytosol of these cells [28,43,97,98,100,101,102,103]. This metabolic signature of quiescent adult stem cells is actively sustained by the following processes and features that are characteristic of these cells: (1) the transcription factor hypoxia-inducible factor 1α (HIF-1α)-dependent upregulation of levels of many glycolytic enzymes (such as hexokinase, phosphofructokinase, glyceraldehyde 3-phosphate dehydrogenase, phosphoglycerate kinase, and enolase) and lactate dehydrogenase A (an enzyme involved in the formation of lactate from pyruvate) in the cytosol; (2) a downregulation of the MPC1 subunit of the pyruvate carrier complex in mitochondria; (3) a HIF-1α-dependent upregulation of the mitochondrial pyruvate dehydrogenase kinases PDK2 and PDK4, both of which inhibit the pyruvate dehydrogenase complex-driven conversion of pyruvate to acetyl CoA in mitochondria; (4) a dynamin-related protein 1 (DRP1)-dependent fragmentation of the mitochondrial network into globular and immature mitochondria with underdeveloped cristae; (5) an increased abundance of the mitochondrial membrane uncoupling protein UCP2 that uncouples and lowers oxidative phosphorylation (OXPHOS) in mitochondria; (6) an elevated concentration of the ATPase inhibitory factor 1 (IF1) that suppresses mitochondrial adenosine triphosphate (ATP) synthase activity; (7) an upregulation of the mitochondrial carrier homolog 2 (MTCH2), a negative regulator of mitochondrial OXPHOS that induces mitochondrial depolarization; (8) an HIF-2α-dependent upregulation of the primary antioxidant enzymes involved in the detoxification of reactive oxygen species (ROS); among these enzymes are catalase (CAT) in peroxisomes and mitochondria, glutathione peroxidase type 1 (GPX1) in peroxisomes and mitochondria, copper/zinc superoxide dismutase (SOD1) in the cytosol, mitochondria, and peroxisomes, and manganese superoxide dismutase (SOD2) in mitochondria; and (9) a decline in the extent of ROS-inflicted apoptotic cell death (Figure 1) [27,43,94,96,97,100,103,104,105,106,107,108,109,110,111,112].

A body of evidence supports the notion that the common features of adult stem cells are to metabolize carbohydrates mainly through aerobic glycolysis in the cytosol, to suppress carbohydrate oxidation in mitochondria, to fragment mitochondrial network into globular and immature mitochondria with underdeveloped cristae, and to stimulate ROS detoxification in several cellular locations are essential for the maintenance of quiescence, identity, high number, regulated proliferation, and controlled differentiation of these cells [43,71,94,95,96,97,100,101,110,113]. Specifically, the HIF-1α, HIF-2α, and transcription factor Meis1 (myeloid ecotropic viral insertion site 1)-dependent program of an intensified glycolytic flow, a weakened mitochondrial OXPHOS, and an enhanced ROS detoxification within hematopoietic stem cells residing in a hypoxic niche is essential for the maintenance of their quiescent state and number (Figure 1) [28,109,110,114,115,116,117,118,119,120,121]. An increased glycolytic flow and a decreased mitochondrial OXPHOS are also required for the quiescent state maintenance and number preservation in mesenchymal stromal cells that are known to reside within the hypoxic environment of the bone marrow niche [28,122].

Mechanisms through which the augmented glycolytic flow in adult stem cells can contribute to the maintenance of the quiescent state and number of these cells by regulating some downstream cellular processes require further investigation. One possibility is that such flow allows the glycolytic intermediate glucose-6-phosphate to enter the pentose phosphate pathway (PPP), which generates nicotinamide adenine dinucleotide phosphate (NADPH) (Figure 1) [123]. NADPH is not only the source of cellular reducing equivalents required for the synthesis of nucleic acids and lipids; it is also the electron donor that is essential for sustaining cellular redox homeostasis via the glutathione reductase system [123]. Since this system protects cellular macromolecules from oxidative damage inflicted by ROS, the intensified glycolytic flow in adult stem cells can defend macromolecules in these cells against ROS-dependent oxidative damage (Figure 1) [96,124,125,126]. The decline in mitochondrial OXPHOS, which is also characteristic of adult stem cells and generates the bulk of ROS, can contribute to the maintenance of the quiescent state and number of these cells through the two mechanisms described in Section 2.3.

### 2.2. Fatty Acid Oxidation and Synthesis Define the Fate of Quiescent Adult Stem Cells

Since mitochondrial transport and the oxidation of pyruvate are actively suppressed in adult stem cells (see above), the transport of fatty acid to mitochondria and the β-oxidation of fatty acids in mitochondria define the rate of mitochondrial respiration in these quiescent cells [43,97].

The efficiencies of mitochondrial fatty acid transport and β-oxidation regulate the self-renewal potential and proliferation capacity of adult stem cells [28,95,97]. A promyelocytic leukaemia protein (PML)/peroxisome proliferator-activated receptor-gamma coactivator 1α (PGC-1α)/peroxisome proliferator-activating receptor type δ (PPARδ)-dependent activation of transcription of nuclear genes involved in mitochondrial fatty acid transport and β-oxidation within hematopoietic stem cells is essential for the self-renewal of these cells by asymmetric divisions, in favor of symmetric differentiating divisions [127,128,129]; this is likely because such activation promotes the mitophagic degradation of damaged and dysfunctional mitochondria (Figure 2) [130].

The self-renewal of neural stem cells by asymmetric divisions also requires the efficiency of mitochondrial fatty acid β-oxidation to reach a certain threshold; it remains unknown why and how such optimal efficiency of mitochondrial fatty acid β-oxidation can fuel asymmetric divisions of neural stem cells but not their symmetric differentiating divisions [95,131,132].

Moreover, an optimal efficiency of mitochondrial fatty acid β-oxidation is needed for the self-renewal of quiescent skeletal muscle stem cells; however, the functionality and viability of these cells are impaired if such efficiency exceeds a certain, oxidative damage-inducing level [95,133,134].

Taken together, these findings indicate that there is a certain optimal efficiency of mitochondrial fatty acid β-oxidation at which this metabolic pathway actively supports the self-renewal potential and proliferation capacity of adult stem cells [95]. Mechanisms underlying this effect of mitochondrial fatty acid β-oxidation are presently unknown. One possible mechanism may involve the demonstrated ability of mitochondrial fatty acid β-oxidation to generate the bulk of NADPH [28,135], which is essential for sustaining cellular redox homeostasis via the glutathione reductase system in adult stem cells (see Section 2.1).

The self-renewal potential and proliferation capacity of adult stem cells can be controlled not only by the efficiencies of mitochondrial fatty acid transport and β-oxidation, but also by the efficiency with which fatty acids are synthesized in the cytosol. Specifically, the acetyl-CoA carboxylase (ACC) and fatty acid synthase (FASN)-dependent synthesis of fatty acids in the cytosol must be inhibited by thyroid hormone-inducible hepatic protein (THRSP; also known as the 14th spot of proteins [SPOT14] protein) to sustain the quiescent state of adult neural stem cells (Figure 2) [136]. This is most likely because certain concentrations of endogenous fatty acids can stimulate the exit of these cells from the quiescent state and their subsequent proliferation and differentiation [28,136]. However, some exogenously added acids and a high-fat diet rich in fatty acids exhibit the opposite effect on the quiescence and proliferation of a different kind of adult stem cell, specifically of intestinal stem cells [137]. In fact, endogenous fatty acids promote the quiescent state of these stem cells, and suppress their proliferation and differentiation [137].

In sum, these findings support the notion that a balance between the mitochondrial β-oxidation of fatty acids and their synthesis in the cytosol defines the fate of adult stem cells, because this balance controls the concentration of fatty acids within these cells. A regulated change of this balance can increase or decrease the intracellular concentration of fatty acids, thereby altering the efficiency with which these cells can sustain quiescence, self-renew by asymmetric divisions, or differentiate. It is also conceivable that different kinds of adult stem cells may differ in the threshold level of fatty acid concentrations that are capable of regulating the fate of these cells.

### 2.3. A Dual Role of ROS in Defining the Homeostasis and Functionality of Quiescent Adult Stem Cells

Since mitochondrial respiration is the major source of ROS within mammalian cells [138], the suppression of mitochondrial respiration and OXPHOS observed in adult stem cells elicits a significant decline in ROS within these cells [96,139,140].

The low concentration of ROS is a hallmark of adult stem cells, and it plays a dual essential role in sustaining the homeostasis, functionality, and long-term survival of these cells [2,71,95,96,97,124,125,126,141,142,143,144,145].

One role of such regulated decline in ROS is to protect the molecules of proteins, lipids, and nucleic acids within adult stem cells (including hematopoietic stems cells, neural stem cells, spermatogonial stem cells, and tracheal stem cells) against oxidative damage inflicted by ROS concentrations exceeding a toxic threshold [71,96,105,146,147,148,149,150]. If ROS concentrations within adult stem cells exceed such a toxic threshold, the DNA and other macromolecules in these cells are oxidatively damaged; this causes cell death, a decline in the number of adult stem cells, and ultimately leads to the exhaustion of the pool of these cells [71,151,152]. The protection of cellular macromolecules against ROS-dependent oxidative damage within adult stem cells is further enhanced by the high efficiencies of the antioxidant defense and DNA repair systems that are characteristic of these cells [96,124,125,126,153,154,155]. The antioxidant defense and DNA repair systems within adult stem cells include the following: (1) the NADPH-dependent glutathione reductase system for sustaining cellular redox homeostasis, which is fueled by the intensified glycolytic flow within these cells, as described in Section 2.1 (Figure 1); (2) the antioxidant system driven by the transcriptional activator FoxO3 of the forkhead family, which is activated by the NAD^+^-dependent protein deacetylase sirtuin 1 (SIRT1); this system includes peroxisomal and mitochondrial CAT, mitochondrial SOD2, and mitochondrial thioredoxin-dependent peroxide reductase (PRDX3) [156,157,158,159,160,161,162]; (3) the antioxidant system orchestrated by the nuclear respiratory factor 2 (NRF2); this system includes enzymes involved in the synthesis of reduced glutathione and thioredoxin, NADPH-generating enzymes of the TCA cycle and pentose phosphate pathway, several forms of PRDX, and cytosolic thioredoxin reductase 1 (TXNRD1) [163,164]; and 4) the non-homologous end-joining (NHEJ) pathway for repairing DNA double-strand breaks [2,165]. In sum, the intensity of oxidative stress in adult stem cells is low. Such intensity is known to be defined by a balance between the efficiencies of cellular ROS production, ROS-inflicted oxidative damage, ROS detoxification, and oxidative damage repair [166,167,168,169]. Thus, the low concentration of ROS within adult stem cells, their high antioxidant capacity, and their enhanced ability to repair oxidatively damaged DNA protect these cells from excessive oxidative stress and damage.

It needs to be emphasized that the FoxO3-dependent and NRF2-dependent antioxidant defense systems, as well as the NHEJ pathway of DNA repair, are indispensable for the quiescence and/or long-term survival of adult stem cells. In fact, the FoxO3-dependent antioxidant defense system is essential for the self-renewal of neural stem cells by asymmetric divisions that allow maintaining a pool of these cells in a quiescent state [157,159,170,171]. Furthermore, the NRF2-dependent antioxidant defense system is required to preserve a pool of intestinal stem cells in the state of quiescence [163,164,172,173]. Moreover, the NHEJ pathway of DNA repair is needed for assuring the long-term survival of adult stem cells [2,174].

Another role of the regulated decline in ROS within quiescent adult stem cells is to suppress their symmetric differentiating divisions. These divisions: 1) are stimulated by ROS concentrations that exceed a certain threshold but remain non-toxic; and 2) cause proliferation and then the terminal differentiation of the cells, thereby exhausting the pool of quiescent adult stem cells [71,96,99,139,140,147,148,150,175,176,177,178]. Such stem cell proliferation-based and differentiation-based exhaustion of the pool of quiescent adult stem cells in response to a rise in ROS concentrations to a level below the toxic threshold has been reported for hematopoietic stems cells, intestinal stem cells, and skeletal muscle stem cells [71,157,159,163,164,170,171,175,179,180,181,182].

The downstream targets of ROS concentrations that elicit the cell proliferation-based and differentiation-based exhaustion of the pool of quiescent adult stem cells remain to be characterized. One of these targets of ROS in hematopoietic stems cells can be the p38α mitogen-activated protein kinase signaling pathway [183,184]. In response to increased ROS concentrations, this pathway stimulates the transcription factor Mitf (microphthalmia-associated transcription factor) that then activates the transcription of a gene for a rate-limiting enzyme of purine metabolism [184,185,186]. The resulting remodeling of purine metabolism alters the levels of amino acids and purine nucleotides, thus promoting cell cycle progression and cell proliferation in response to increased ROS concentrations [184,185,186].

### 2.4. Some Mitochondrial TCA Cycle Intermediates Contribute to Quiescence Maintenance by Adult Stem Cells

Recent findings suggest that the suppression of mitochondrial respiration and OXPHOS is not the only mitochondria-related factor involved in sustaining the homeostasis and functionality of adult stem cells. Specifically, it has been shown that some intermediates of the mitochondrial TCA cycle play essential roles in determining the fate of these cells; these intermediates include acetyl CoA, oxaloacetate, citrate, and α-ketoglutarate [31,77,94,187,188,189,190,191].

The essential roles of these TCA cycle intermediates in the fate of adult stem cells is due to their abilities to be cofactors or substrates for chromatin-modifying enzymes that catalyze some epigenetic modifications in the nucleus, such as histone acetylation/deacetylation and histone methylation, as well as DNA methylation and hypermethylation [2,31,71,77,94,187,188,189,190,191,192,193,194,195,196]. Some of these mitochondrial metabolism-driven modifications of histones and DNA have been observed only in neural stem cells, skeletal muscle stem cells, adipose-derived mesenchymal stem cells, muscle-derived mesenchymal stem cells, and bone marrow-derived mesenchymal stem cells, but not in their fully differentiated progeny; such stem cell-specific epigenetic modifications in the nucleus play essential roles in maintaining quiescence, regulating self-renewal and proliferation, and/or suppressing the differentiation of these adult stem cells [2,31,77,133,197,198,199,200,201,202,203,204,205,206,207,208,209,210,211,212].

Many of these stem cell-specific epigenetic modifications in the nucleus are known to activate or repress the transcription of genes implicated in different aspects of fate programming in adult stem cells, including the maintenance of their quiescence and self-renewal ability as well as their regulated proliferation and differentiation [2,31,77,188,189,197,198,199,200,201,202,203,206,207,210]. Nuclear genes whose transcription is activated in quiescent neural stem cells, muscle stem cells, hair follicle stem cells, umbilical cord-derived mesenchymal stem cells, and bone marrow-derived mesenchymal stem cells include genes that encode proteins that are involved in the G1/S cell cycle transition, epigenetic modifications in the nucleus, transcription of genes implicated in stem cell fate decisions, stemness, and microRNA formation [2,189,213,214,215,216]. Nuclear genes whose transcription is repressed in quiescent neural stem cells, muscle stem cells, hair follicle stem cells, adipose-derived mesenchymal stem cells, muscle-derived mesenchymal stem cells, and bone marrow-derived mesenchymal stem cells include genes that encode proteins implicated in DNA replication, chromosome segregation, cell cycle delay at several checkpoints, cell cycle progression, lineage specificity, and mitochondrial functionality [2,188,213,214,215,216].

Taken together, these findings suggest that certain mitochondrial metabolism-driven modifications of the epigenetic landscape within the nucleus of adult stem cells can alter the efficiencies with which some transcription factors activate or repress the expression of nuclear genes that are involved in the quiescent state maintenance by adult stem cells and/or in their response to environmental cues, proliferation, and differentiation [2,31,77,78,94,96].

Further investigation is required on the mechanisms through which the TCA cycle intermediates acetyl CoA, oxaloacetate, citrate, and α-ketoglutarate can maintain the homeostasis and functionality of adult stem cells by promoting epigenetic modifications of nuclear genes and altering the epigenetic landscape of these cells. In hematopoietic stems cells, skeletal muscle stem cells, and hair follicle stem cells, the histone 3 methyltransferases EZH1 and EZH2 (enhancer of zeste proteins 1 and 2, respectively) are essential components of such mechanisms; in fact, the downregulation of both proteins leads to a depletion of the pool of these quiescent adult stem cells, whereas the upregulation of EZH2 decelerates the exhaustion of hematopoietic stems cells [217,218,219,220].

### 2.5. NAD^+^ Concentration within Adult Stem Cells Defines Their Fate

Relative rates of aerobic glycolysis, lactate formation, mitochondrial respiration, OXPHOS, mitochondrial TCA cycle, and mitochondrial fatty acid β-oxidation control the cellular concentrations of the oxidized and reduced forms of nicotinamide dinucleotide (NAD^+^ and NADH, respectively) and, thus, the NAD^+^/NADH ratio in adult stem cells [94,96,221]. The NAD^+^/NADH ratio plays an essential role in defining the fate of adult stem cells not only because NAD^+^ is a cofactor of all these metabolic processes, but also because NAD^+^ is an activator of SIRT1 in the cytosol and SIRT3 in mitochondria [222,223,224].

A body of evidence supports the essential role of NAD^+^-activated SIRT1 in defining the fate of adult neural stem cells. However, there are two conflicting views on how exactly NAD^+^-activated SIRT1 can influence the fate of these cells. On the one hand, some studies suggest that NAD^+^-activated SIRT1 promotes the quiescence of adult neural stem cells and suppresses their proliferation and differentiation. These studies have shown that (1) the genetic inactivation or pharmacological inhibition of SIRT1 in adult neural stem cells promotes their exit from quiescence and stimulates their proliferation and differentiation [225,226,227,228,229], and (2) SIRT1 overexpression or its resveratrol-driven activation preserves the quiescence of adult neural stem cells and suppresses their proliferation and differentiation [226,229]. On the other hand, other studies have suggested that NAD^+^-activated SIRT1 suppresses the quiescence of adult neural stem cells and promotes their proliferation and differentiation. These other studies have demonstrated that (1) SIRT1 genetic downregulation or pharmacological inhibition promotes the quiescence of these cells and suppresses their proliferation and differentiation; and (2) SIRT1 overexpression stimulates the quiescence exit of these cells and accelerates their proliferation and differentiation in a mechanism that involves a transient translocation of SIRT1 from the cytosol into the nucleus [230,231,232]. In sum, the exact role of NAD^+^-activated SIRT1 in defining the fate of adult neural stem cells remains controversial and requires further investigation.

The essential role of NAD^+^ concentration and SIRT1 activity in defining the fate of adult stem cells is underscored by the finding that a decline in NAD^+^ concentration within skeletal muscle stem cells lowers SIRT1 activity; this causes an increase in the extent of histone H4 acetylation and ultimately activates the transcription of nuclear genes involved in the transition from quiescence to the proliferation and differentiation of these stem cells [133,233]. Thus, one role of the NAD^+^-dependent protein deacetylase SIRT1 in maintaining the quiescence of adult skeletal muscle stem cells is linked to its known ability to suppress the transcription of certain genes by specifically remodeling their epigenetic landscape through histone H4 deacetylation (Figure 3) [234,235,236].

Another mechanism through which NAD^+^-activated SIRT1 contributes to quiescence maintenance in adult neural stem cells and adult skeletal muscle stem cells consists of the SIRT1-dependent deacetylation and activation of PGC-1α (a transcriptional activator of mitochondrial biogenesis) to promote mitochondrial functionality (Figure 3) [75,237,238,239].

It needs to be emphasized that the exact role of NAD^+^-activated SIRT1 in the quiescence of adult skeletal muscle stem cells requires further investigation. Specifically, it has been demonstrated that when SIRT1 is activated by NAD^+^ in these stem cells, it promotes autophagy by deacetylating and stimulating the autophagy-related protein 7 (ATG7) to provide the energy and macromolecules required for the transition from quiescence to proliferation followed by differentiation (Figure 3) [240,241]. This finding suggests that under certain conditions, NAD^+^-activated SIRT1 can act via an autophagy-based mechanism that stimulates the exit from quiescence and promotes the proliferation and differentiation of adult skeletal muscle stem cells.

Of note, it has been proposed that NAD^+^-activated SIRT1 in hematopoietic stem cells and neural stem cells may help to sustain their quiescent state by deacetylating another quiescence-related target of SIRT1, namely the DNA-binding forkhead box O (FOXO) transcription factors [48]. The SIRT1-dependent deacetylation of the FOXO transcriptional factors is known to enhance their ability to activate the transcription of many genes that are essential for preserving the quiescence of hematopoietic stem cells and neural stem cells, preventing their premature differentiation and maintaining their viability (Figure 3) [48,157,159,170,171,242,243].

SIRT3, a mitochondrial form of the NAD^+^-dependent sirtuins, is essential for the maintenance of quiescence in adult hematopoietic stem cells [46,244,245]. Since NAD^+^-stimulated SIRT3 deacetylates and activates some mitochondrial proteins involved in oxidative stress protection, it contributes to the quiescence maintenance of these cells by diminishing oxidative stress inside and outside of their mitochondria [46,244,245]. Specifically, when activated by NAD^+^, SIRT3 deacetylates and activates SOD2, which is a major mitochondrial antioxidant enzyme; this reduces mitochondrial superoxide and weakens cellular oxidative stress (Figure 3) [245,246,247]. NAD^+^-stimulated SIRT3 also deacetylates and activates isocitrate dehydrogenase 2 (IDH2) [244,248]. Mitochondrial IDH2 catalyzes a reaction that yields NADPH, the electron donor that is essential for sustaining cellular redox homeostasis via the glutathione reductase and thioredoxin reductase systems [123]. Thus, the SIRT3-driven deacetylation and activation of IDH2 also lessens cellular oxidative stress (Figure 3) [244,248].

### 2.6. A Low-Energy Status of Adult Stem Cells Helps Sustain Their Quiescence

As noted above, low efficiencies of OXPHOS and ATP synthesis in mitochondria are characteristic features of the metabolic signature of quiescent adult stem cells [94,96,97]. This causes a rise in the intracellular concentrations of adenosine monophosphate (AMP) and adenosine diphosphate (ADP), thereby creating a low-energy stress and activating the liver kinase B1 (LKB1; a master energy-sensing protein kinase) and the AMP-activated protein kinase (AMPK; a downstream phosphorylation target of LKB1) [249,250,251,252].

LKB1 is essential for the quiescence maintenance, functionality, and survival of hematopoietic stem cells in mice. In fact, a depletion of LKB has the following two temporally separated effects on hematopoietic stem cells and on the multipotent progenitors formed from these adult stem cells: (1) an initial loss of stem cell quiescence and the resulting rise in the number of cells comprising each of these two cell populations; and (2) a subsequent decline in the number, and the ultimate depletion, of both cell populations [253,254,255]. The hematopoietic stem cells of mice depleted of LKB1 lost the ability to regenerate the hematopoietic system of control mice, and this decline in the functionality of LKB1-depleted hematopoietic stem cells is followed by their apoptotic death [253,254,255]. A downstream target of LKB1 in hematopoietic stem cells is mitochondrial functionality, as LKB1 depletion in these cells decreases the expression of the transcriptional activators of mitochondrial biogenesis PGC-1α and PGC-1β, lowers mitochondrial ATP synthesis, and impairs mitochondrial integrity by decreasing mitochondrial membrane potential [253,254,255]. The decline in the integrity of mitochondria observed within LKB1-depleted hematopoietic stem cells is likely to be responsible for their accelerated apoptotic death [254]. These cells also undergo an induction of autophagy, which plays an essential protective role in the survival of LKB1-depleted hematopoietic stem cells [254]. It is conceivable that the ability of LKB1 to sustain the functionality and integrity of mitochondria, thereby decelerating apoptotic cell death, is linked to the essential role of this master energy-sensing protein kinase in preserving the quiescence, functionality, and survival of hematopoietic stem cells [253,254,255,256]. The involvement of LKB1 in preserving the quiescence of hematopoietic stem cells is complemented by the essential role of autophagy—a degradative elimination of dysfunctional macromolecules and organelles—in sustaining the quiescent state of these adult stem cells [257]. Although AMPK—the downstream phosphorylation and activation target of LKB1—is known to promote autophagy because it phosphorylates the autophagy-initiating protein kinase ULK1 [252]—a mechanism through which autophagic degradation is actively stimulated and sustained in hematopoietic stem cells to preserve their quiescence—it also requires further investigation.

The essential role of LKB1 in sustaining the quiescence, functionality, and survival of hematopoietic stem cells is not due to its activating effect on AMPK in these cells [253,254,255]. AMPK is a sensor of energetic stress and mitochondrial dysfunction that does not influence the fate of hematopoietic stem cells, neither before nor after being phosphorylated and activated by LKB1 [253,254,255,258]. Phosphorylated and activated AMPK is known to suppress the mammalian (or mechanistic) target of rapamycin complex 1 (mTORC1) signaling pathway [48,252], whose genetic enhancement elicits a depletion of hematopoietic stem cells [259,260,261,262,263,264] (see Section 2.7 for more details). Despite such an essential role of a low-intensity mTORC1 signaling in maintaining hematopoietic stem cell quiescence, the indispensable role of LKB1 in sustaining the quiescence, functionality, and survival of hematopoietic stem cells is not caused by the LKB1-driven and AMPK-dependent activation of the mTORC1 signaling pathway [253,254,255,258].

Although LKB1 controls the fates of hematopoietic stem cells and the multipotent progenitors formed from these cells, it does not affect the abundance or functionality of the fully differentiated progeny of such cells [253,254,255]. In contrast, LKB1 is not required for sustaining the quiescence, functionality, and survival of a different type of adult stem cells, namely intestinal stem cells [265]. However, LKB1 is essential for the normal differentiation and maturation of intestinal stem cells; mechanisms underlying such effects of LKB1 are presently unknown [265,266,267]. Thus, LKB1 can differently influence the self-renewal, proliferation, and differentiation of different types of adult stem cells.

### 2.7. The Maintenance of mTORC1 Signaling at Low Intensity in Adult Stem Cells Is Essential for Sustaining Their Quiescence, Self-Renewal, and Functionality

The mTORC1 signaling pathway is a key signaling hub that—in response to changes in extracellular and intracellular nutrients, pro-mitogenic stimuli, and stresses—alters the efficiencies of many anabolic and catabolic cellular processes to ensure a proper adaptation of the cell to such changes [268,269]. The anabolic processes controlled by mTORC1 include glycolysis and the pentose phosphate pathway, lipid and nucleotide synthesis, and protein synthesis [268,269]. Among the catabolic processes controlled by mTORC1 are autophagy and lysosome biogenesis, and the ubiquitin-dependent proteasomal degradation of proteins [268,269]. mTORC1 also controls cellular energy homeostasis by activating the YY1 (yin-yang 1)/PGC-1α-dependent transcription of nuclear genes involved in mitochondrial biogenesis and functionality [270,271,272].

Since some of the cellular processes regulated by mTORC1 can be involved in creating the metabolic signature of adult stem cells that is essential for their quiescence (see Section 2.1, Section 2.2, Section 2.3, Section 2.4, Section 2.5 and Section 2.6), and because some of the processes creating such signatures are known to control mTORC1 via feedback regulation mechanisms [28,270,271,272], it is conceivable that mTORC1 may play an essential role in defining the fate of quiescent adult stem cells. In fact, an overactivation of the mTORC1 pathway by a mutation that depletes the protein inhibitor Pten (phosphatase and tensin homologue) of this pathway causes an exit of hematopoietic stem cells from quiescence, leads to a decline in functionality of these cells, decreases their self-renewal potential, alters their differentiation pattern, and eventually results in an exhaustion of the hematopoietic stem cell population pool in a cell-autonomous, mTORC1-dependent fashion (Figure 4) [259,260]. Furthermore, an enhancement of the mTORC1 pathway by a constitutive expression of the upstream protein activator Akt1 (RAC-alpha serine/threonine protein kinase 1 or v-akt murine thymoma viral oncogene homolog 1) of mTORC1 transiently increases the abundance of hematopoietic stem cells; then, it causes their excessive proliferation, and ultimately elicits the apoptotic death of hematopoietic stem cells and the resulting depletion of their pool in an mTORC1-dependent manner (Figure 4) [263,264]. Moreover, an overstimulation of the information flow through the mTORC1 pathway by a mutation that conditionally deletes the upstream protein inhibitor Tsc1 (tuberous sclerosis 1) of mTORC1 promotes an exit of hematopoietic stem cells from the quiescent state, impairs their functionality and self-renewal ability, promotes their apoptotic death, and eventually depletes the pool of hematopoietic stem cells in an mTORC1-dependent fashion (Figure 4) [261,262]. These effects of Tsc1 deletion on the fate of hematopoietic stem cells (1) coincide with a rise in the abundance of mitochondria and a rise in cellular ROS; and (2) can be reversed with the help of the antioxidant *N*-acetylcysteine, a ROS antagonist [261]. Thus, the maintenance of mTORC1 signaling at low intensity in hematopoietic stem cells is essential for sustaining their quiescence, because it allows the suppression of the YY1/PGC-1α-dependent transcription of nuclear genes involved in mitochondrial biogenesis and functionality [270]. Such suppression prevents a rise of mitochondrially-produced cellular ROS above a threshold level that is capable of inducing the quiescence exit and proliferation of these adult stem cells (Figure 4) [261].

The intensity of information flow through the mTORC1 signaling pathway also defines the fate of a different type of quiescent adult stem cells, namely neural stem cells, as well as the neural progenitor cells that are formed from them. In fact, a mutation that conditionally deletes Tsc1 to enhance this pathway impairs the migration patterns of both neural stem cells and neural progenitor cells in the subventricular zone of the brain [273]. Moreover, a downregulation of the mTORC1 signaling pathway by caloric restriction in Paneth cells of the intestinal stem cell niche triggers a cell non-autonomous mechanism that promotes the self-renewal of intestinal stem cells and improves their functionality [274]. Thus, the fate of intestinal stem cells also depends on the intensity of information flow through the mTORC1 signaling pathway. Downstream cellular processes whose regulation by mTORC1 controls quiescence maintenance, self-renewal, and functionality of neural stem cells and intestinal stem cells are presently unknown.

The nature of the intracellular pro-mitogenic and/or anti-mitogenic signals whose ability to control mTORC1 in a cell-autonomous manner defines the fate of quiescent adult stem cells requires further investigation. Recent studies uncovered a cell-nonautonomous mechanism of such mTORC1 control in muscle stem cells. Specifically, it was demonstrated that the abilities of muscle stem cells to respond to injury-induced systemic signals from distant tissues by retaining a pool of quiescent stem cells and by creating a population of differentiated cells for distant tissue repair are under tight control of the mTORC1 signaling within these adult stem cells [20,275,276]. Tissue injury stimulates the circulating protease hepatocyte growth factor activator (HGFA); then, HGFA relays a signal to muscle stem cells in tissues distant to the zone of injury by proteolytically activating HGF to stimulate the HGF receptor cMet (mesenchymal–epithelial transition factor) and promote mTORC1 signaling in these stem cells via a presently unknown mechanism (Figure 4) [20,275,276]. Once promoted, mTORC1 orchestrates a transition of adult stem cells from the G_0_ phase to the “alert” (G_Alert_) phase of quiescence; this increases the cell size, mitochondrial activity, and ATP concentration and, ultimately commits these stem cells to cell cycle entry, proliferation, and differentiation (Figure 4) [20,275,276].

### 2.8. Several Mechanisms of Proteostasis Maintenance in Quiescent Adult Stem Cells Define Their Fate

As discussed in Section 2.3, an essential role of ROS in defining the homeostasis and functionality of quiescent adult stem cells consists in the ability of ROS concentrations that exceed a toxic threshold to inflict oxidative damage to cellular proteins. Several mechanisms for maintaining protein homeostasis (proteostasis) within these cells are known to play essential roles in preserving the quiescence, self-renewal, proliferation capacity, differentiation potential, and long-term survival of adult stem cells. Some of these mechanisms prevent a collapse of cellular proteostasis by regulating protein synthesis and folding, whereas other mechanisms are involved in the proteasomal or autophagic degradation of damaged and dysfunctional proteins and organelles [29,277,278,279,280]. These mechanisms are integrated into a proteostatic network orchestrated by several signaling pathways and transcriptional factors that define the fate of adult stem cells [29,278,279]. The common traits of the proteostatic network operating in adult stem cells include the following: (1) a suppression of protein synthesis on free ribosomes in the cytosol; (2) a rise in the concentrations of heat shock proteins (HSPs) that act as chaperones or co-chaperones to assist the proper folding and stability of newly synthesized proteins; (3) an activation of the unfolded protein response (UPR) systems in the endoplasmic reticulum (ER) and mitochondria (UPR^ER^ and UPR^mit^, respectively); (4) an enhancement of the ubiquitin system for the ubiquitin/proteasome-dependent proteolytic clearance of improperly folded proteins, cell-cycle regulators, and transcriptional factors, as well as for the proteasome-independent regulation of surface protein receptors, histones, ribosome assembly factors, and vesicle transport proteins; and (5) a stimulation of autophagy, a quality control mechanism for the regulated lysosomal degradation of dysfunctional or excessive proteins and organelles, in some types of adult stem cells or its maintenance at a basal level in other types of such cells (Figure 5 and Figure 6) [29,79,277,278,279,280,281,282,283,284]. The involvement of each of these proteostatic mechanisms in the homeostasis and functionality of adult stem cells is outlined below in this section.

#### 2.8.1. Protein Synthesis on Free Ribosomes in the Cytosol

The rate of protein synthesis in hematopoietic stem cells is significantly lower than that in differentiating progenitor cells formed from them, which is likely because the repressor 4EBP1 (eukaryotic translation initiation factor 4E-binding protein 1) of cap-dependent translation is less phosphorylated and more active in these adult stem cells [285]. The protein synthesis rate that is maintained in hematopoietic stem cells is optimal for sustaining their quiescence and self-renewal potential. In support of this notion, it was shown that (1) a mutation that increases such a rate within hematopoietic stem cells in an mTORC1-dependent manner depletes their pool; (2) a mutation that decreases the protein synthesis rate within hematopoietic stem cells weakens their self-renewal ability; and (3) a combination of these two mutations restores the “optimal” protein synthesis rate within hematopoietic stem cells and salvages the above effects of both mutations on stem cell quiescence and self-renewal [259,285]. Therefore, the 4EBP1-dependent decline in protein synthesis rate observed in hematopoietic stem cells is required for the maintenance of their homeostasis and functionality. The efficiency of ribosome assembly in hematopoietic stem cells is an essential contributing factor to such an indispensable role of the “optimal” protein synthesis rate in defining the fate of these adult stem cells. In fact, a mutation in the ribosome protein Rpl22l impairs the emergence of hematopoietic stem cells [286]. Furthermore, a depletion of the transcription factor Runx1 in hematopoietic stem cells weakens ribosome biogenesis, decelerates protein synthesis, decreases cell susceptibility to apoptotic death, and makes these adult stem cells more resistant to genotoxic and ER stresses [287]. Moreover, a mutation that impairs the maturation and nuclear export of the pre-60S ribosomal subunit in hematopoietic stem cells causes an exhaustion of the pools of quiescent cells and the multipotent progenitors formed from them, but not of the pool of mature hematopoietic cells [288]. In addition, a decline in ribosome abundance within hematopoietic stem cells suppresses the translation of a specific set of mRNAs that are essential for erythroid lineage commitment after these stem cells exit the quiescent state [289].

Akin to hematopoietic stem cell quiescence, self-renewal, and differentiation, the fate of neural stem cells also depends on the rate of mTORC1-regulated protein synthesis. Specifically, the constitutive activation of 4EBP1 (which represses cap-dependent translation) in neural stem cells with hyperactive mTORC1 and protein synthesis exceeding the optimal rate has been shown to restore the optimal rate of protein synthesis, increase self-renewal potential, and slow down the accelerated differentiation of these adult stem cells [290].

Protein synthesis on free ribosomes in the cytosol also defines the homeostasis and functionality of epidermal stem cells, as a mutation that impairs the recycling and rescue of ribosomes stalled before protein synthesis completion has been demonstrated to increase the synthesis rates of all the cellular proteins in an mTORC1-dependent manner, cause the excessive proliferation of these adult stem cells, and alter their normal differentiation pattern [291]. Since a suppression of mTORC1 signaling by rapamycin in epidermal stem cells carrying this mutation decreases the global protein synthesis rate and salvages the above effects of the mutation of the fate of epidermal stem cells, the maintenance of a protein synthesis rate below a certain threshold is essential for sustaining the homeostasis and functionality of these adult stem cells [291].

The fate of skeletal muscle stem cells depends on the synthesis rates of myogenic factor 5 (Myf5) and myoblast determination protein (MyoD) that are translated from a distinct set of mRNA templates. In quiescent skeletal muscle stem cells, the translation of these mRNAs is selectively suppressed either by a protein kinase R (PKR)-like endoplasmic reticulum kinase (PERK)-dependent phosphorylation of translation initiation factor eIF2α [292] or by their association with certain microRNAs [293,294]. This causes an accumulation of such mRNAs in cytoplasmic mRNP (messenger ribonucleoprotein) granules, thus silencing their translation [292,293,294]. Such silencing is essential for the maintenance of a reversible quiescent state by skeletal muscle stem cells [292,293,294]. Furthermore, the dissociation of the cytoplasmic mRNP granules caused either by an eIF2α dephosphorylation or by a downregulation of the microRNAs allows the translation of these mRNAs in skeletal muscle stem cells, and is required for both their exit from quiescence and their entry into a differentiation program [292,293,294].

#### 2.8.2. HSP Concentrations

Many HSPs are chaperones or co-chaperones that assist the proper folding and stability of newly synthesized proteins [295,296]. The concentrations of some HSPs in various types of quiescent adult stem cells exceed those in the fully differentiated progeny of such cells [279,297]. Specifically, the abundance of HSP70 protein 5 (HSPA5), HSP70 protein 8 (HSPA8), and HOP (an HSP70-HSP90 organizing protein) in neural stem cells and mesenchymal stem cells is higher than in their differentiated progeny [298]. Adipose-derived stem cells exhibit higher concentrations of HSP27 (HSPB1), αB-crystallin (HSPB5), HSP20 (HSPB6), and HSP60 than the fully differentiated progeny of such cells [299].

Some of these HSPs play essential roles in preserving the quiescence, abundance, survival, proliferation capacity, and differentiation potential of adult stem cells, as outlined below.

Mechanisms underlying such effects of some HSPs have begun to emerge. HSC70 (HSPA8) is essential for the cytokine-mediated survival of hematopoietic stem cells and prevents their differentiation; this is because this HSP decreases the stability of mRNA encoding BIM, which is a BH3-only pro-apoptotic factor that compromises stem cell viability but is required for apoptosis during hematopoiesis and leukemogenesis (Figure 5) [300]. HSC70 (HSPA8) also interacts with cyclin D1 and cyclin-dependent kinase inhibitors p27 and p57 in the cytosol of hematopoietic stem cells [301]. Both p27 and p57 prevent the nuclear import of the HSC70/cyclin D1 complex, thereby allowing the maintenance of quiescence of hematopoietic stem cells (Figure 5) [301]. The GRP78 member of the HSP70 protein family binds to teratocarcinoma-derived growth factor 1 (TDGF-1) on the surface of hematopoietic stem cells, and this binding allows maintaining the quiescent state of these adult cells via the TDGF-1-dependent induction of glycolysis (Figure 5) [302]. HSP70 (HSPA5) indirectly stimulates the erythroid differentiation of hematopoietic stem cells because it prevents the caspase-3-mediated proteolysis of GATA sequence protein 1 (GATA-1), which is a transcriptional factor that is essential for the terminal differentiation of erythroid progenitors formed from these adult stem cells (Figure 5) [303]. The HSP mortalin in the mitochondria of hematopoietic stem cells interacts with the antioxidant deglycase protein 1(DJ-1), which is implicated in Parkinson’s disease, and the mortalin/DJ-1 complex allows sustaining hematopoietic stem cell quiescence, abundance, and self-renewal capacity, because it protects the mitochondria of these cells from ROS accumulation and oxidative macromolecular damage (Figure 5) [304].

An overexpression of αB-crystallin (HspB5), a small HSP that is essential for muscle development and homeostasis [305,306], slows down the differentiation of skeletal muscle stem cells; this effect of αB-crystallin (HspB5) is due to its ability to decelerate synthesis and accelerate the degradation of MyoD, which is a master protein regulator of such differentiation (Figure 5) [307]. Moreover, HSP70 (HSPA5) is indispensable for the osteogenic and chondrogenic differentiation of human mesenchymal stem cells, perhaps because it enhances the expression of the bone morphogenetic protein 2 member of the transforming growth factor β(TGF-β) superfamily of secreted polypeptide factors [308,309].

#### 2.8.3. The UPR^ER^ and UPR^mit^ Systems

If the HSPs-assisted folding of newly synthesized proteins is not enough to sustain their proper conformations, the build-up of misfolded and unfolded proteins in the ER and mitochondria activates the UPR^ER^ and UPR^mit^ systems (respectively) to refold or degrade these proteins and restore cellular proteostasis [310,311,312,313].

When activated, the UPR^ER^ system allows restoring proteostasis in the ER, because it decelerates protein synthesis to decrease protein flow to the ER, promotes a refolding of some improperly folded proteins accumulated in this organelle, and directs other improperly folded proteins amassed in the ER for degradation by ER-associated degradation (ERAD) or autophagy [310,311,314]. If these protective processes that are integrated into the UPR^ER^ system are unable to reinstate proteostasis in the ER, a coordinated action of the ER and mitochondria triggers the mitochondria-controlled apoptotic death of the entire cell [315].

When the UPR^mit^ system is activated, it enables proteostasis reinstatement in mitochondria because it stimulates the refolding of some improperly folded proteins that accumulated in these organelles with the help of mitochondrial chaperone systems and because it also promotes the proteolytic degradation of other improperly folded proteins with the help of proteolytic systems in mitochondria [312,313,316,317].

As outlined below, both the UPR^ER^ and UPR^mit^ systems of proteostasis restoration are indispensable for maintaining the quiescence, self-renewal, proliferation capacity, differentiation potential, and functionality of adult stem cells.

The RNA binding protein Dppa5 (developmental pluripotency-associated 5 protein), the tauroursodeoxycholic bile acid, and hypoxia-inducible factor 2α (HIF-2α) stimulate the UPR^ER^ system to elicit a decline in ER stress within hematopoietic stem cells and protect these cells from apoptotic death caused by excessive ER stress [318,319,320]. Such stimulation of the UPR^ER^ system is required for the maintenance, self-renewal, functionality, and survival of hematopoietic stem cells (Figure 6) [318,319,320]. If ER stress in the quiescent hematopoietic stem cells of humans exceeds a certain threshold and cannot be resolved by the UPR^ER^ system, these cells commit suicide by undergoing apoptotic death [321]. This prevents the propagation of dysfunctional quiescent hematopoietic stem cells that amass improperly folded and aggregated proteins in the ER [321]. Progenitor cell populations that are formed from these human quiescent hematopoietic stem cells following their activation can avoid an ER stress-induced apoptotic death, because they undergo an adaptive enhancement of the UPR^ER^ system and can resolve ER stress [321]. The UPR^ER^ system is also indispensable for the lymphopoietic and erythropoietic differentiation programs of primed mouse hematopoietic stem cells; however, mechanisms underlying such effects of the UPR^ER^ system require further investigation [322,323,324].

Of note, the UPR^ER^ system does not always define the fate of hematopoietic stem cells, because under certain conditions, ER stress can be resolved with the help of chemical compounds assisting in maintaining proteostasis within the ER. Specifically, bile acids secreted from maternal and fetal liver act as chemical chaperones that prevent the accumulation of improperly folded and aggregated proteins in the ER, thereby decreasing ER stress and allowing the expansion of a population of hematopoietic stem cells during hematopoiesis in the fetal liver of mice [325].

The protein kinase PERK of the UPR^ER^ phosphorylates the eukaryotic translation initiation factor 2α (eIF2α) in mouse skeletal muscle stem cells, thus suppressing the translation of many mRNAs that lack upstream open reading frames (uORFs) in their 5′ untranslated regions [292]. mRNAs whose translation is suppressed by eIF2α phosphorylation in these adult stem cells encode the proteins that are needed for quiescence exit followed by proliferation and myogenic differentiation, whereas mRNAs with uORFs whose translation is not suppressed by such phosphorylation encode proteins involved in quiescence maintenance (Figure 6) [292]. Mutations that prevent the PERK-dependent eIF2α phosphorylation impair the abilities of skeletal muscle stem cells to maintain quiescence and self-renew, while an inhibitor of eIF2a dephosphorylation increases the self-renewal potential of these cells and improves their functionality [292]. Thus, the PERK arm of the UPR^ER^ system controls the quiescence, self-renewal, differentiation, and functionality of skeletal muscle stem cells in mice. The activation of this arm of the UPR^ER^ system following muscle injury in mice is also essential for the survival of progenitor cells formed from primed skeletal muscle stem cells and for the differentiation of these progenitor cells during regenerative myogenesis and muscle formation [326].

In the nucleus of mouse quiescent hematopoietic stem cells, the histone deacetylase SIRT7 is very abundant and interacts with the transcription factor nuclear respiratory factor 1 (NRF1), which in its free form activates the transcription of genes encoding mitochondrial ribosomal proteins and translation factors [327]. The interaction between NRF1 and SIRT7 impairs the ability of NRF1 to activate the transcription of these genes, thus inhibiting mitochondrial translation and biogenesis, lowering mitochondrial protein stress, and suppressing the UPR^mit^ system (Figure 6) [327]. Such SIRT7-driven suppression of the UPR^mit^ system is essential for maintaining the quiescence and differentiation potential of hematopoietic stem cells [327].

Pharmacological interventions that rise the intracellular concentration of NAD^+^ in mice have been shown to stimulate the protein deacetylase SIRT1, which then increases mitochondrial functionality and activates the UPR^mit^ system by increasing the abundance of two members of the prohibitin protein family of mitochondrial stress sensors and effectors (Figure 6) [75,239]. Such NAD^+^/SIRT1-dependent activation of the UPR^mit^ system (including prohibitins) is indispensable for the quiescence, self-renewal, differentiation, and viability of muscle stem cells, neural stem cells, and melanocyte stem cells in mice (Figure 6) [75,239].

#### 2.8.4. The Ubiquitin System

The enhancement of the ubiquitin system characteristic of quiescent adult stem cells is sometimes considered only as a mechanism for the ubiquitin/proteasome-dependent proteolytic clearance of improperly folded proteins whose proper conformations in these stem cells cannot be restored by their HSPs-assisted folding and/or by their UPR^ER^ and UPR^mit^-dependent refolding or degradation [29,278,279]. However, the ubiquitin system is known to play essential proteolysis-related and proteolysis-unrelated roles in preserving the quiescence, self-renewal, proliferation capacity, and differentiation potential of adult stem cells, not because it helps to clear improperly folded proteins, but mainly because it regulates the stability, conformation, activity, localization, protein-binding specificity, or vesicular trafficking of properly folded cell-cycle regulators, surface protein receptors, transcription factors, histones, ribosome assembly factors, and vesicle transport proteins that define the fate of these stem cells [281,283,284].

The specificity with which ubiquitin is covalently attached to certain lysine residues of the proteins that define the fate of adult stem cells depends on many E3 ubiquitin ligases, each ubiquitinating a distinct set of protein targets [284,328]. The ubiquitin E3 ligases c-Cbl, Itch, Fbw7, Skp2, and Huwe1 are indispensable for sustaining quiescence and preventing the excessive self-renewal and overproliferation of hematopoietic stem cells, thus precluding a depletion of this adult stem cell population and impeding an exhaustion of hematopoiesis [29,281,283,284,329,330,331,332,333,334,335,336,337,338,339,340,341,342,343,344,345,346]. This is because these ubiquitin E3 ligases ubiquitinate and prime for proteasome degradation a distinct group of proteins that are essential for maintaining hematopoietic stem cell homeostasis by regulating signal transduction, the transcription of many nuclear genes, cell growth and proliferation, cell cycle progression, and mitochondria-controlled apoptosis; among these proteins are Notch1, c-Kit, STAT5, c-Myc, n-Myc, cyclin E, p27, and Mcl-1 [29,281,283,284,329,330,331,332,333,334,335,336,337,338,339,340,341,342,343,344,345,346]. A depletion of the ubiquitin E3 ligases c-Cbl, Itch, Fbw7, Skp2 and Huwe1 in hematopoietic stem cells elicits their loss of quiescence, enhances their self-renewal, increases their abundance, prompts their overproliferation, decelerates their differentiation, and eventually causes a depletion of the hematopoietic stem cell population [29,281,283,284,340,343,347,348,349,350,351].

When hematopoietic stem cells move out from their hypoxic niche in the low-oxygen environment of the bone marrow to enter the differentiation program, the ubiquitin E3 ligase VHL (von Hippel–Lindau) ubiquitinates the hypoxia-inducible transcription factor HIF-1α, thus promoting its proteasomal degradation [116,352]. HIF-1α is a master controller of the transcription program for sustaining the quiescence, functionality, and survival of the hematopoietic stem cells sustained within the low-tension oxygen niche [117,120,353]. The VHL-driven proteasomal degradation of HIF-1α in the hematopoietic stem cell niche is required for the migration of these cells from the niche and for the ensuing exit of these cells from quiescence and entry into differentiation [116]. In fact, VHL depletion increases the stability of HIF-1α, promotes the quiescence of hematopoietic stem cells and their early progenitors, and impairs the proliferation and differentiation of these cells during hematopoiesis [116].

In adult neural stem cells, the ubiquitin E3 ligase APC/C (anaphase-promoting complex) controls a balance between the cell quiescence maintenance program and the programs for cell quiescence exit, proliferation, and differentiation, because it primes for proteasome degradation or non-proteolytically activates certain cell-cycle regulation proteins, transcription factors, translation factors, and cell surface receptor proteins [284,354,355,356,357,358,359]. HUWE1 (HECT, UBA and WWE domain containing protein 1), another ubiquitin E3 ligase in adult neural stem cells, stimulates a re-entry of early neural progenitor cells into the quiescent state, because it ubiquitinates and primes some transcription factors for proteasome degradation, cell-cycle regulation proteins, and apoptotic death regulators [284,360,361,362,363,364].

In sum, the above findings indicate that the ability of ubiquitin E3 ligases to prime for proteasome degradation (or, in some cases, to activate in a non-proteolytic manner) a distinct set of proteins in different types of adult stem cells is essential for controlling a balance between the processes of cell quiescence maintenance, exit, and re-entry. As it has been reviewed elsewhere, the ubiquitin E3 ligases-dependent proteasome degradation is also indispensable for regulated self-renewal, which is a response to signals emerging from the niche, proliferation, differentiation, functionality and survival of various adult stem cells [281,283,284].

#### 2.8.5. Autophagy

Autophagy, a quality control mechanism for regulated lysosomal degradation of damaged or dysfunctional proteins and organelles, is required for sustaining the quiescent state, self-renewal ability, proliferation capacity, differentiation potential, fitness, functionality, and/or long-term survival of quiescent adult stem cells [29,78,79,278,279,282,365,366].

In different types of adult stem cells, the activity of autophagy is either higher or lower than that in the differentiating progenitor cells formed from them [29,78,79,278,279,282,365,367].

Autophagy activity in hematopoietic, dermal, epidermal, and mesenchymal stem cells exceeds that in progenitor cells derived from these adult stem cells [368,369]. Such a rise in autophagic activity within adult hematopoietic stem cells is due to the induced expression of many pro-autophagy genes, which is driven by the forkhead transcription factor FoxO3 [369,370,371]. The increase of autophagic activity within hematopoietic stem cells is essential for sustaining their quiescent state, self-renewal ability, differentiation potential, high resistance to stresses, fitness, functionality, and viability [257,368,369,371,372,373,374,375]. In fact, mutations eliminating several key protein components of autophagic machinery impair these essential features of hematopoietic stem cells [257,368,369,371,372,373,374,375]. These mutations increase the abundance of mitochondria, stimulate mitochondrial ROS production, enhance ROS-inflicted oxidative damage to cellular components, and accelerate mitochondria-controlled apoptotic cell death [257,371,372,373,374,375]. Therefore, it is believed that the FoxO3-induced autophagy is essential for the quiescence, self-renewal, differentiation, stress resistance, fitness, functionality, and viability of hematopoietic stem cells, because it selectively eliminates functional and dysfunctional mitochondria to suppress the excessive formation of ROS in these organelles, lower oxidative cellular damage, and prevent mitochondria-controlled cell death [257,371,372,373,374,375].

Autophagy activity in neural stem cells and cardiac stem cells is lower than that in the progenitor cells formed from these adult stem cells; such activity further rises during the differentiation of both types of progenitor cells [376,377,378]. Mutations eliminating the Atg5 and Ambra1 protein components of autophagic machinery in neural stem cells and the pharmacological interventions that suppress autophagy in both these types of adult stem cells impair early steps of stem and progenitor cell differentiation [376,377,378]. In contrast, pharmacological interventions that stimulate autophagy in cardiac stem cells promote the differentiation of these stem cells and their early progenitors [377,378]. It is conceivable that autophagy activation in progenitor cells formed from neural stem cells and cardiac stem cells is required to fulfill the high-energy demands of the differentiation process [376,377,378]. Of note, akin to Atg5 and Ambra1 depletions, a depletion of the autophagy-inducing protein FIP200 impairs the differentiation of early progenitors of neural stem cells [379]. Since such an FIP200-dependent impairment of neural stem cell progenitors can be salvaged with the help of an anti-oxidant chemical compound, autophagy activation in these progenitor cells may not only provide energy to fuel the differentiation process, but may also suppress excessive oxidative damage to cellular components during differentiation [379]. FIP200 depletion also causes a progressive depletion of the pool of neural stem cells because it triggers their apoptotic death [379]. Thus, the basal level of autophagy activity observed in these adult stem cells plays an essential role in protecting them from the apoptotic death caused by excessive cellular stress.

Similar to the autophagy activity in neural stem cells and cardiac stem cells, such activity in the muscle stem cells of young mice is lower than the autophagy activity in the progenitor cells that are derived from them [365,367]. The basal level of autophagy activity in these muscle stem cells is essential for the establishment and maintenance of their quiescence state, as a depletion of the Atg7 protein component of autophagic machinery in these cells impairs the reversible G_0_ state of quiescence, decreases stem cell number and functionality, and promotes entry into the irreversible G_0_ state of senescence [365,367]. The entry of autophagy-deficient muscle stem cells into the irreversible senescent state is caused by a build-up of dysfunctional mitochondria, an accumulation of ROS in excessive concentrations, a rise in oxidative damage to cellular components, and a resulting decline of proteostasis in these cells [365,367]. The accumulation of excessive ROS in autophagy-deficient muscle stem cells is responsible for their entry into the irreversible senescent state; in fact, an anti-oxidant chemical compound that decreases cellular ROS has been shown to prevent such senescence entry and restore the self-renewal of autophagy-deficient muscle stem cells [365,367].

In addition to the essential role of autophagy in establishing and maintaining the quiescence of muscle stem cells, autophagy is also indispensable for the exit of muscle stem cells from the quiescent state, the activation of their myogenic differentiation, and the progression through several stages of such differentiation and muscle regeneration. Specifically, during the transition of muscle stem cells from the quiescent state to the activation state, autophagy activity rises in a SIRT1-dependent manner to provide the nutrients and ATP needed for the energy-demanding process of entry into myogenic differentiation [240]. Furthermore, in response to muscle injury, autophagy is stimulated in early progenitors of muscle stem cells, and the extent of such autophagy stimulation defines the efficiency of muscle regeneration via a presently unknown mechanism [380]. Moreover, autophagy is also involved in myotube formation during muscle regeneration, but the mechanism of such involvement remains to be investigated [381].

### 2.9. Cell Cycle Regulatory Proteins Are Essential for Sustaining the Quiescence, Self-Renewal, Functionality, and Differentiation Potential of Adult Stem Cells

Several cell cycle regulatory proteins are integrated into a network of cell-intrinsic mechanisms that define the fate of quiescent adult stem cells [2,4,382,383,384,385,386,387].

One of these proteins is the tumor suppressor protein p53, which is a transcription factor that can arrest the cell cycle at the G_1_/S checkpoint because in response to DNA damage, it activates the expression of the cyclin-dependent kinase Cdk2 inhibitor p21 [388,389,390]. A depletion of p53 in hematopoietic stem cells, which promotes quiescence exit and cell cycle entry, enhances self-renewal and raises the number of fully functional hematopoietic stem cells [391,392,393,394]. Thus, p53 is essential for sustaining the quiescent state of hematopoietic stem cells and inhibiting their proliferation and self-renewal. The essential role of p53 in sustaining the pool of hematopoietic stem cells at a certain level is independent of p21 [394] but requires Necdin, which is a growth-suppressing protein whose expression is activated by p53 at the transcriptional level [395]. The ability of p53 to fully sustain the functional hematopoietic stem cells by promoting the apoptotic death of those of them that build-up excessive DNA damage is under the control of Aspp1, which is a member of the family of apoptosis-stimulating proteins of p53 [396].

p53 also suppresses the proliferation and self-renewal of neural stem cells and promotes the apoptotic death of those of them that are dysfunctional (likely because they amass DNA damage). In fact, p53 depletion in neural stem cells stimulates their proliferation and mitigates their apoptotic death [397]. The mechanisms underlying these effects of p53 in neural stem cells are presently unknown.

Three members of the retinoblastoma protein family (pRBs) are tumor suppressor proteins that, in their hypophosphorylated forms, can arrest the cell cycle at the G_1_/S checkpoint, because they are transcriptional repressors of genes that are essential for G_1_/S transition [398,399]. If pRBs are hyperphosphorylated by different cyclin/cyclin-dependent kinase complexes in response to certain pro-mitogenic stimuli, they unable to suppress cell cycle entry at the G_1_/S checkpoint [399,400]. As outlined below, similar to p53, pRBs also define the fate of different types of quiescent adult stem cells.

A simultaneous depletion of all the pRBs in adult hematopoietic stem cells elicits the exit of these stem cells from quiescence, impairs the reconstitution ability of these stem cells in a transplantation assay, causes an excessive proliferation of both stem cells and their early hematopoietic progenitors, and promotes apoptosis in lymphoid (but not in myeloid) progenitor populations [401]. Hence, a collective action of pRBs is essential for sustaining the quiescence of hematopoietic stem cells, preventing their unbalanced proliferation, maintaining their transplantation functionality, and retaining an unbiased differentiation of the lymphoid and myeloid cell lineages in the hematopoietic system.

A depletion of a single member of the pRBs family in muscle stem cells causes their permanent exit from the quiescent state, accelerates cell cycle re-entry in both stem cells and their progenitor myoblast cells (thus substantially expanding these two cell populations), and significantly decelerates terminal differentiation into myotubes; these effects of the depletion of a single pRB member deteriorate muscle fiber formation, slow muscle growth, and delay muscle repair [402]. Thus, even a partial decline in the abundance of pRBs alters the fate of muscle stem cells.

Among the cell cycle regulatory proteins that define the fate of quiescent adult stem cells are several cyclin-dependent kinase inhibitors. Their essential roles in maintaining the functionality of adult stem cells are discussed below.

As mentioned in Section 2.8.2, the interaction of cyclin-dependent kinase inhibitors p27 and p57 with the HSC70/cyclin D1 complex in the cytosol of hematopoietic stem cells prevents the nuclear import of this protein complex [301]. A depletion of both p27 and p57 in mice permits the nuclear import of the HSC70/cyclin D1 complex, thereby stimulating pRB hyperphosphorylation by the complex formed between cyclin D1 and cyclin-dependent kinases Cdk4/6 in the nucleus and impairing the ability of pRB to arrest the cell cycle of hematopoietic stem cells at the G_1_/S checkpoint [301]. Due to these effects, mouse hematopoietic stem cells depleted of both p27 and p57 fail to maintain quiescence, are decreased in number, have low self-renewal activity, and exhibit a loss of the reconstitution ability in a transplantation assay [301]. Thus, a collective action of p27 and p57 is essential for sustaining the quiescence, self-renewal, and functionality of hematopoietic stem cells.

A depletion of the cyclin-dependent kinase 2 (Cdk2) inhibitor p21 in mice impairs the abilities of hematopoietic stem cells and neural stem cells to sustain quiescence, lowers their self-renewal potentials, reduces their reconstitution abilities in a transplantation assay, decreases their numbers, and ultimately causes an exhaustion of their pools [403,404]. Hence, p21 is indispensable for the maintenance of the quiescence, self-renewal ability, and fitness in both hematopoietic stem cells and neural stem cells.

## 3. Conclusions

In this review, we compared many metabolic, signal transduction, gene expression, epigenetic, stress survival, cell cycle regulation, and other traits of different types of mammalian and human adult stem cells. Our comparison indicates that adult stem cells have evolved an intricate network of cell-intrinsic mechanisms that control the establishment and preservation of these traits. These mechanisms regulate quiescence entry, maintenance, and exit in response to certain extrinsic pro-mitogenic and anti-mitogenic cues from the microenvironment of adult stem cells. One important challenge is to understand how the numerous cell-intrinsic mechanisms of quiescence entry, maintenance, and exit are integrated in space and time within mammalian and human adult stem cells. The other challenge is to decipher the hierarchical order and relative contributions of the different traits of adult stem cells in the ability of these cells to enter, sustain, and exit quiescence in response to specific cell-extrinsic factors within different tissue-specific microenvironments. Future work will also aim at understanding how the history of spatial and temporal changes in cell growth and division conditions is translated into the pattern of quiescence entry, maintenance, and exit in different types of adult stem cells. Since the impairment of a balance between the quiescence, proliferation, and differentiation of adult stem cells has been implicated in the pathophysiology of many diseases of old age, addressing these challenges in the future will increase our understanding of how this balance can be controlled to delay cellular and organismal aging and postpone the onset of aging-associated diseases.
